# Candidate protein biomarkers in chronic kidney disease: a proteomics study

**DOI:** 10.1038/s41598-024-64833-8

**Published:** 2024-06-18

**Authors:** Zhalaliddin Makhammajanov, Assem Kabayeva, Dana Auganova, Pavel Tarlykov, Rostislav Bukasov, Duman Turebekov, Mehmet Kanbay, Miklos Z. Molnar, Csaba P. Kovesdy, Syed Hani Abidi, Abduzhappar Gaipov

**Affiliations:** 1 Department of Biomedical Sciences, School of Medicine, Nazarbayev University, Astana, Kazakhstan; 2 Department of Internal Medicine, Astana Medical University, Astana, Kazakhstan; 3 Department of Proteomics and Mass Spectroscopy, National Center for Biotechnology, Astana, Kazakhstan; 4 Department of Chemistry, School of Sciences and Humanities, Nazarbayev University, Astana, Kazakhstan; 5 Division of Nephrology, Department of Internal Medicine, Koc University, Istanbul, Turkey; 6 Division of Nephrology & Hypertension, Department of Internal Medicine, Spencer Fox Eccles School of Medicine at the University of Utah, Salt Lake City, UT USA; 7 Division of Nephrology, Department of Medicine, University of Tennessee Health Science Center, Memphis, TN USA; 8 Department of Medicine, School of Medicine, Nazarbayev University, Astana, Kazakhstan; 9 Clinical Academic Department of Internal Medicine, University Medical Center, Astana, Kazakhstan

**Keywords:** Biomarkers, Chronic kidney disease, Proteinuria, Urinary proteomics, Biomarkers, Biomarkers, Chronic kidney disease, Chronic kidney disease

## Abstract

Proteinuria poses a substantial risk for the progression of chronic kidney disease (CKD) and its related complications. Kidneys excrete hundreds of individual proteins, some with a potential impact on CKD progression or as a marker of the disease. However, the available data on specific urinary proteins and their relationship with CKD severity remain limited. Therefore, we aimed to investigate the urinary proteome and its association with kidney function in CKD patients and healthy controls. The proteomic analysis of urine samples showed CKD stage-specific differences in the number of detected proteins and the exponentially modified protein abundance index for total protein (p = 0.007). Notably, specific urinary proteins such as B2MG, FETUA, VTDB, and AMBP exhibited robust negative associations with kidney function in CKD patients compared to controls. Also, A1AG2, CD44, CD59, CERU, KNG1, LV39, OSTP, RNAS1, SH3L3, and UROM proteins showed positive associations with kidney function in the entire cohort, while LV39, A1BG, and CERU consistently displayed positive associations in patients compared to controls. This study suggests that specific urinary proteins, which were found to be negatively or positively associated with the kidney function of CKD patients, can serve as markers of dysfunctional or functional kidneys, respectively.

## Introduction

Chronic kidney disease (CKD) is one of the leading global health challenges, imposing a substantial burden on society. Recent studies show that around 10% of people in developed countries are affected by CKD, mainly people over 65 years of age, due to comorbidities and age-related factors^1,2^. Over the last three decades, CKD-related mortality has increased by almost 1% yearly, becoming a serious global health issue^3,4^. Unfortunately, the disease is projected to become the fifth leading cause of death by 2040^5^. This is a concerning trend that highlights its impact on global mortality rates. Contributing factors to the increase in CKD-related mortality include the aging population and the rise of CKD-causing diseases, including diabetes and arterial hypertension^6^. Also, late diagnosis of CKD and the financial burden associated with initiating treatments at advanced stages exacerbate CKD progression, leading to end-stage kidney disease (ESKD) and cardiovascular complications.

Therefore, early detection and intervention are essential to address these challenges. Lifestyle modifications and medications, such as SGLT2 inhibitors and renin–angiotensin–aldosterone modulators, have shown promise in preserving kidney function^7^.

Notably, biomarkers, particularly proteinuria, are crucial in identifying and managing CKD. The levels of proteinuria serve as a marker for disease progression and associated outcomes in non-diabetic and diabetic individuals^8^. Higher proteinuria levels often correlate with rapid CKD progression^9^. However, clinical observations indicate that some patients with high proteinuria experience a slower decline in kidney function compared to those with lower levels of urinary protein excretion. This variability suggests that the toxic properties of the filtered proteins, rather than just the quantity, play a significant role in influencing the disease progression^10,11^. For example, filtered albumin has been reported to promote cellular reactive oxygen species-mediated tubulointerstitial inflammation and endothelin-1-mediated tubulointerstitial fibrosis^11^. Thus, identifying potential toxic urinary proteins associated with kidney function and understanding its pathophysiological mechanisms are crucial for early CKD detection^11^.

Urine proteomics holds promise in understanding CKD. It offers insights into dynamic protein changes and potential biomarkers for disease identification. Previous proteomic investigations have revealed numerous potential biomarkers for CKD. However, these studies frequently focused on analyzing blood proteins^12^ or spot urine samples^13,14^, potentially limiting their representativeness.

Therefore, the urinary proteome extracted from 24-h urine samples was analyzed in this study. This methodology provides a more comprehensive and stable representation of the dynamic proteome changes associated with CKD disease. Our study aimed to compare the urinary protein profiles of CKD patients with stages 1–3 and healthy participants. The objective was to identify potential biomarkers for clinical use and deepen our understanding of CKD's progression via molecular mechanisms.

## Materials and methods

### Patients and study design

In this cross-sectional study, we enrolled 88 patients with CKD and 49 age-matched healthy controls. Participants with CKD were identified during hospital admission and/or nephrology consultation at the National Scientific Medical Center (NSMC, Astana, Kazakhstan) and were recruited between March 2020 and December 2022. Age-matched healthy controls were volunteers recruited at the same hospital setting after advertising the invitation to this research study.

We included individuals aged ≥ 18 and ≤ 70 years old with CKD stages 1–3, categorized by eGFR according to KDIGO 2012 guidelines^15^. Diagnosis relied on eGFR, kidney damage markers, and clinical evaluation due to the unavailability of kidney biopsies. Kidney function was assessed using the serum creatinine-based eGFR via the 2021 CKD-EPI (CKD Epidemiology Collaboration) equation^16^. Control participants had no clinical or laboratory indicators of CKD, hypertension, or other known diseases. Exclusions comprised age outside the 18–70 range, eGFR < 60 mL/min for controls, and eGFR < 30 mL/min for patients, presence of acute infections, cancer, and pregnancy.

Written informed consent was obtained from all the study participants. This study was approved by the Nazarbayev University Institutional Review Ethics Committee (NU-IREC 208/06122019) and registered in ClinicalTrials.gov as a part of a clinical trial (ID NCT04311684)^17^. Based on good medical and laboratory practice, all the principles of the Declaration of Helsinki for Biomedical Research Involving Human Participants were met during patient examinations.

### Laboratory tests

Sample collection procedures were conducted independently of patient prognoses. All laboratory personnel involved were blinded to the clinical outcomes. Blood samples were used for complete blood count and biochemical analyses, assessing various metabolic parameters such as glucose, lipids, urea, creatinine, uric acid, and total protein.

Moreover, 24-h urine samples were used for biochemical analysis to determine total protein levels. The blood and urine clinical laboratory analyses were performed using a colorimetric method on a COBAS Integra 400 plus analyzer (Roche Diagnostics, Indianapolis, Indiana, United States) at the NSMC.

The remaining urine samples were stored at − 80 °C and subsequently utilized for proteomics analysis at the National Center for Biotechnology (NCB) in Astana. The proteomics process involved extracting protein from 24-h urine samples using an acetone precipitation method^18^. Proteins were resuspended and stored at − 80 °C before concentration measurement with a NanoDrop 1000 (Thermo Scientific, Waltham, Massachusetts, United States). In-solution protein digestion was performed with protein concentrations ranging from 30 to 50 μg.

### Mass spectrometry analysis

Urinary proteins underwent trypsin digestion (20 ng/μL) at 37 °C overnight following reduction and alkylation. Peptide mixtures were purified and concentrated using a ZipTip with 0.6 μL C18 resin (Millipore, Burlington, Massachusetts, United States). Eluted peptides were processed with a centrifugal evaporator (Eppendorf, Hamburg, Germany), resuspended in 16 μL of 0.1% trifluoroacetic acid, and 15.5 μL of the sample was loaded into a liquid chromatography-tandem mass spectrometry (LC–MS/MS) machine.

Chromatography was performed using a Dionex HPLC pump with an Acclaim PepMap100 C18 pre-column and Acclaim PepMap100 C18 RSLC column (Thermo Scientific, Waltham, Massachusetts, United States). The samples were analyzed using a nanoflow reversed-phase C18 LC–MS/MS instrument. The Impact II ESI-QUAD-TOF mass spectrometer (Bruker Daltonics, Bremen, Germany) with a whole captive spray ion source was utilized for analyzing digested urinary proteins, operating at parameters such as dry temperature 150 °C, dry gas 3.0 L/min, capillary 1500 V.

Full-scan MS spectra were obtained at a 2.0 Hz spectral rate, followed by one MS/MS spectrum. Data Analysis 3.4 software (Bruker Daltonics, Bremen, Germany) was used to analyze the retrieved MS/MS data, saved in Mascot generic format (*.mgf).

Proteins and peptides were identified using Mascot 2.6.1 software (Matrix Science, London, UK) against the Swiss-Prot database (release February 2021), which was taxonomically restricted to *Homo sapiens*. The following parameters were applied to the search in the Mascot software: carbamidomethylation of cysteine residues as the fixed modification and oxidation of methionine as the variable modification; the maximum missed cleavages of tryptic peptides were set to two; peptides and proteins were identified with two significance thresholds: *P* < 0.05 and *P* < 0.001. The decoy database search was used for the false discovery rate (FDR) estimation, and the FDR was set to 1%. Allowed mass error windows for MS and MS/MS were 100 ppm and 0.05 Da, respectively. The Exponentially Modified Protein Abundance Index (emPAI) was used for label-free peptide quantification^19^.

The proteomic dataset underwent log2-transformation and average and slope-based normalization steps to ensure data consistency and integrity for subsequent analyses.

### Gene Ontology (GO) enrichment analysis

The significant GO terms associated with negatively and positively correlated proteins with eGFR, adjusted for proteinuria, were identified using the org.Hs.eg.db^20^ and clusterProfiler^21^ packages in R. Proteins from both correlation and regression analyses were considered. The enrichGO function was used for the enrichment analysis, categorizing negatively and positively associated proteins based on their involvement in biological processes, cellular components, and molecular functions. The FDR cutoff of 0.05 was considered.

### Reactome pathway analysis

The pathways associated with negatively and positively correlated proteins with eGFR, adjusted for proteinuria, were identified using the ReactomePA package^22^ in R. Proteins from both correlation and regression analyses were considered. The enrichPathway function was used to identify significantly enriched pathways with *P* value < 0.05 and an adjusted *q* value < 0.05.

### Protein–protein interaction analysis

STRING-DB (version 12)^23^ was utilized to explore protein–protein interactions. The corresponding gene identifiers of negatively and positively correlated proteins with eGFR, adjusted for proteinuria, were input into the STRING web platform, enabling the exploration of known and predicted protein interactions from various sources, including experimental data, co-expression, and text mining.

### Data visualization

The visualization was created using the ggplot2 package^24^ in R. A heatmap was generated to represent the presence of proteins in control and CKD 1–3 groups. Also, volcano plots were generated to visualize the relationship between eGFR and urinary proteins, depicting significance levels and coefficients. The top enriched GO terms and Reactome pathways were also visualized. Bar plots were generated to demonstrate the most significantly enriched biological processes and pathways for up and downregulated protein sets. The results were reported in -log10 scale to enhance visual clarity.

### Statistical analyses

Statistical analyses were performed using Stata MP2 18. Normally distributed numeric variables were presented as mean ± standard deviation (SD) and non-normally distributed variables as the median and interquartile range (IQR). A two-sided *t*-test and Wilcoxon rank-sum test were used to analyze parametric and non-parametric data between CKD patients and healthy control group (CG). Moreover, patients were categorized into three CKD stage groups; therefore, one-way ANOVA and Kruskal–Wallis tests were used to analyze parametric and non-parametric data between multiple groups. A chi-square test was used to analyze categorical variables, and they were reported as numbers and percentages. Furthermore, Spearman’s correlation test was used to identify the association of the urinary proteome with kidney function (i.e., eGFR). In addition, linear regression analysis was carried out using eGFR and urinary proteome data to determine the influence of proteins on kidney function. Furthermore, regression analysis between eGFR and urinary proteome was performed, adjusting for the amount of 24-h urine protein.

### Prior presentation

Parts of this study were presented at the ISN WCN 2022 and HUPO 2023 Congresses.

## Results

### Clinical and biochemical characteristics of study population

In our study, we categorized participants into CKD risk groups and stages 1, 2, and 3 (Supplementary Table 1). Both groups were age-matched, with mean ages of 38.6 years (SD = 12.3) for patients and 37.2 years (SD = 7.9) for the control group. The patient group included 48% females and 52% males, while the control group included 69% females and 31% males. Analysis of clinical and biochemical parameters at different stages of CKD showed marked differences between the CKD groups as compared to the control group (Table 1). Notably, metabolic markers, including serum urea and glucose, showed significant variations (*P* < 0.001 and *P* = 0.001, respectively), emphasizing the systemic impact of CKD on metabolic homeostasis. Proteinuria was higher in patients with more advanced stages of CKD (Table 1).

**Table 1:** Clinical and biochemical characteristics of participants.

Parameters	CG, *n* = 49	CKD stage 1, *n* = 40		CKD stage 2, *n* = 24		CKD stage 3, *n* = 24	*P-*value
Demographics							
Age, year	36 (31–42)	33 (25–44)		39 (32–50)		41 (36–49)	0.065
Gender, female, n (%)	34 (69)	24 (60)		7 (29)		7 (29)	0.001
eGFR, mL/min/1.73 m^2^	110 ± 13	116 ± 14		72 ± 9		45 ± 10	< 0.001
CKD etiology							
Glomerular, n (%)		32 (80)		16 (67)		17 (71)	
Transplant, n (%)		1 (3)				3 (12)	
Diabetic, n (%)		1 (3)		1 (4)		1 (4)	
Lupus, n (%)		2 (5)		1 (4)			
CKD of unknown etiology, n (%)		4 (10)		6 (25)		3 (12)	
Comorbidities							
Hypertension, n (%)	4 (8)	15 (37)		9 (37)		10 (42)	
Anemia, n (%)	2 (4)	5 (12)		2 (8)		8 (33)	
Laboratory data							
WBC 10 × 10^9^/L	5.8 (5.1–6.4)	7.4 (5.6–11.6)		7 (5.7–9.8)		7.2 (6.2–8.9)	< 0.001
PLT 10 × 10^9^/L	240.9 ± 58.4	306.6 ± 70.8		266.6 ± 71.1		265.4 ± 60.9	< 0.001
RBC 10 × 10^12^/L	4.7 ± 0.5	4.5 ± 0.5		4.7 ± 0.8		4.5 ± 0.8	0.205
HGB g/L	137 ± 17.9	130.9 ± 19.6		138 ± 24.2		128.3 ± 24.6	0.239
ESR, mm/h	8 (5–12)	24 (14–35)		19 (7–30)		18.5 (11–41)	< 0.001
Serum total protein, g/L	69.8 ± 3.4	58.9 ± 10.1		60.7 ± 11.2		57.9 ± 15.1	< 0.001
Total cholesterol, mmol/L	4.7 (4.2–5.1)	6.3 (4.1–7.2)		6 (4.8–7.1)		5.7 (5–6.5)	< 0.001
Serum creatinine, µmol/L	63.9 (55.3–71.8)	61.5 (49.3–69.9)		106.6 (94.9–124.4)		155.3 (139.7–169.7)	< 0.001
Serum uric acid, µmol/L	308.3 ± 93.8	314.8 ± 86.4		414.5 ± 86.4		475.9 ± 122.4	< 0.001
Total bilirubin, µmol/L	7.8 (6.2–12.3)	6.6 (4.6–11.1)		8.2 (3.4–12.6)		5.3 (3.2–7.8)	0.037
Serum urea, mmol/L	4.3 (3.7–4.9)	4.2 (3.5–5.4)		7 (5.7–9.3)		9.9 (8.5–11.5)	< 0.001
Serum glucose, mmol/L	5.1 (4.9–5.6)	4.8 (4.4–5.3)		4.9 (4.5–5.4)		5.4 (5.1–5.7)	0.001
Urinalysis of 24-h urine samples							
Proteinuria, g/24-h	0.1 (0.1–0.1)	1.3 (0.5–3.1)		2 (0.8–3.2)		3.3 (0.9–6.4)	< 0.001
Proteomic data of 24-h urine samples							
Detected proteins, *n*	446	360		251		202	
emPAI for total protein	41.7 (18.8–54.8)	61.4 (35.6–80.7)		49.8 (30.3–101.7)		63.6 (39.5–89.8)	0.007

Normally distributed numeric variables are expressed as mean ± SD and non-normally distributed variables as median (IQR).

*CG* control group, *CKD* chronic kidney disease group, *eGFR* estimated glomerular filtration rate, *emPAI* exponentially modified protein abundance index.

**Figure 1:**
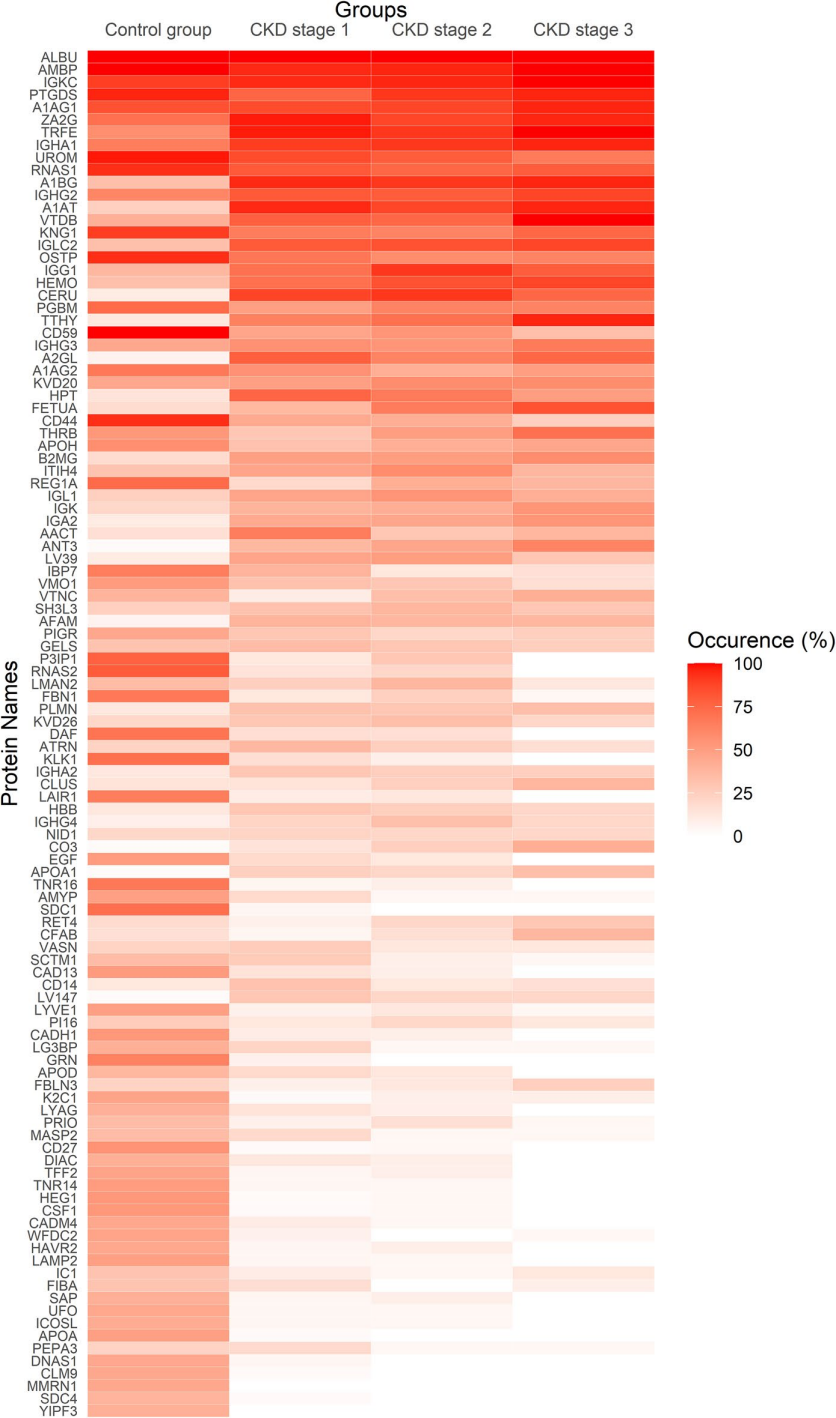
Heatmap of distribution rate of proteins in the control and patient groups*.* Each row on the Y-axis corresponds to a specific protein, while the X-axis columns represent different groups. The color intensity in each heatmap cell reflects the percentage of protein occurrence within the respective sample group, with higher values and color intensity indicating a higher detection rate in that group. CKD = chronic kidney disease.

The proteomic analysis of urine samples revealed significant differences in the number of detected proteins and exponentially modified protein abundance index (emPAI) for total protein between the control and patient groups (*P* = 0.007; Table 1). Additionally, marked differences in the distribution of different protein types were observed across the control and CKD groups (Fig. 1). The number of detected proteins was higher in healthy controls compared to the CKD group, and a lower number of proteins was observed in patients with more advanced stages of CKD (Table 1 and Fig. 1).

### Urinary proteins correlated with kidney function

The correlation analysis between urinary proteome and kidney function showed that some urinary proteins were correlated with eGFR among all study participants (Table 2). In particular, proteins FETUA, B2MG, IGK, KVD20, IGL1, VTDB, and IGHA1 exhibited negative correlations, while proteins IC1, CLUS, VMO1, VTNC, CD59, REG1A, KNG1, LV39, CD44, SH3L3, OSTP, A1AG2, CERU, RNAS1, UROM, and PTGDS were positively correlated with eGFR. While statistically significant, these correlations were predominantly moderate in magnitude.

**Table 2:** Correlations of emPAI of proteins with eGFR in whole cohort.

Urinary proteins	Spearman’s Rho	*P*-value	N of obs	Molecular weight (Da)
FETUA	− 0.441	** < 0.001**	59	39
B2MG	− 0.357	**0.008**	54	14
IGK	− 0.307	**0.032**	49	23
KVD20	− 0.286	**0.018**	68	12
IGL1	− 0.27	**0.048**	54	23
VTDB	− 0.268	**0.01**	93	53
IGHA1	− 0.192	**0.042**	112	43
IC1	0.535	**0.009**	23	55
CLUS	0.498	**0.008**	28	52
VMO1	0.485	** < 0.001**	48	21
VTNC	0.431	**0.005**	41	54
CD59	0.425	** < 0.001**	87	14
REG1A	0.42	** < 0.001**	61	19
KNG1	0.406	** < 0.001**	102	72
LV39	0.401	**0.009**	42	12
CD44	0.362	**0.001**	78	81
SH3L3	0.359	**0.022**	41	10
OSTP	0.325	**0.001**	101	35
A1AG2	0.282	**0.014**	76	24
CERU	0.266	**0.017**	80	122
RNAS1	0.241	**0.01**	114	18
UROM	0.234	**0.011**	116	70
PTGDS	0.191	**0.036**	121	21

Significant values are in bold.

Then, correlation analysis was conducted separately in CKD and control participants to identify differences between the two groups (Table 3). In CKD patients, FETUA, B2MG, AMBP, and VTDB proteins were negatively correlated, while LV39, CD59, A1BG, and CERU were positively correlated with eGFR levels. Among control participants, ATRN, SAP, and LG3BP protein were negatively correlated, and PGRP1 and CRNN were positively correlated with eGFR. The average emPAI values of identified proteins between the two groups are depicted in Supplementary Table 2.

**Table 3:** Correlations of emPAI of proteins with eGFR by two groups.

Urinary proteins	CKD group			Control group			Molecular weight (Da)
	Spearman’s Rho	*P*-value	N of obs	Spearman’s Rho	*P*-value	N of obs	
FETUA	− 0.501	** < 0.001**	50	− 0.417	0.26	9	39
B2MG	− 0.445	**0.002**	45	− 0.483	0.185	9	14
AMBP	− 0.244	**0.026**	84	0.131	0.374	48	39
VTDB	− 0.230	**0.049**	73	− 0.024	0.919	20	53
LV39	0.402	**0.014**	37	− 0.6	0.278	5	12
CD59	0.396	**0.013**	39	0.199	0.174	48	14
A1BG	0.328	**0.003**	83	− 0.198	0.474	15	54
CERU	0.32	**0.005**	75	− 0.5	0.382	5	122
ATRN	0.222	0.295	24	− 0.691	**0.021**	11	158
SAP	1.0	0.024	4	− 0.63	**0.005**	19	58
LG3BP	− 0.173	0.606	11	− 0.572	**0.009**	20	65
PGRP1	0.1	0.869	5	0.566	**0.045**	13	22
CRNN	− 1.0	0.317	2	0.56	**0.048**	13	53

Significant values are in bold.

### Association of urinary proteome with kidney function

**Figure 2:**
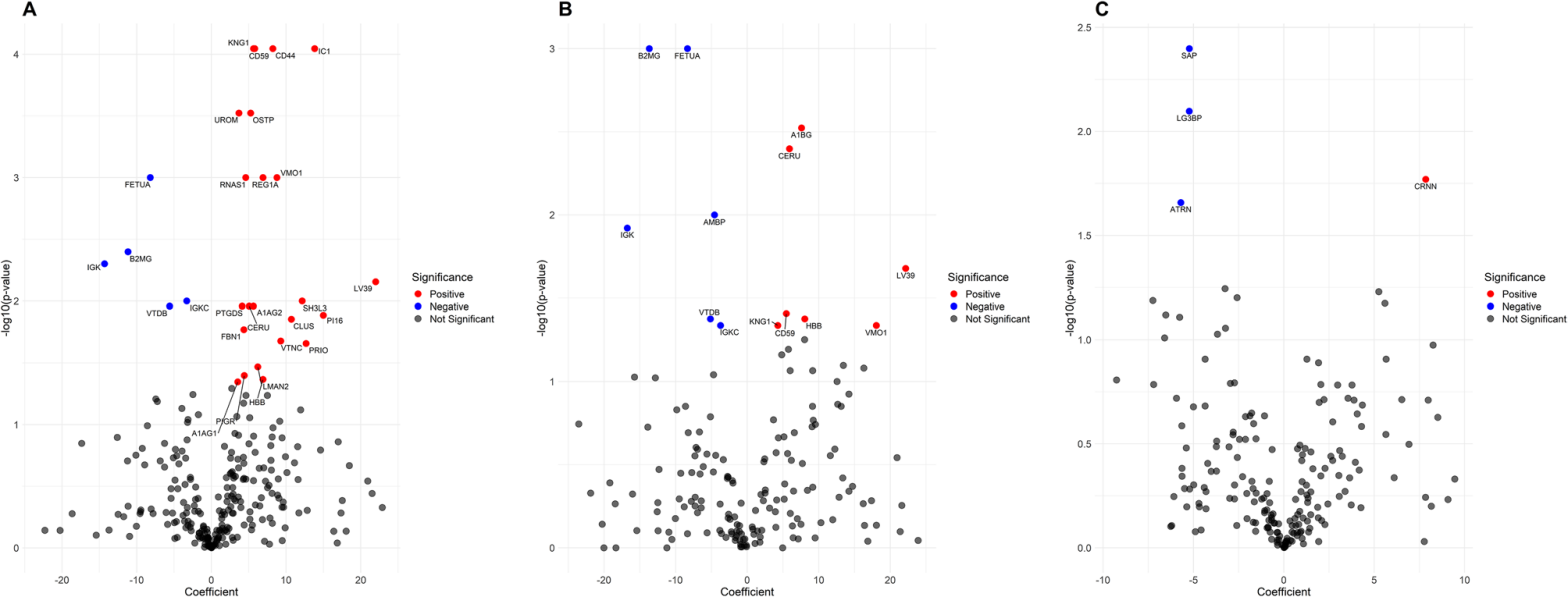
Volcano plot showing association between eGFR and emPAI of proteins. Regression analysis in all participants (**A**) and in patient (**B**) and control groups (**C**). On the X-axis, coefficient values show the relationship of individual proteins with eGFR, while the Y-axis conveys -log10-transformed *P* values for proteins with adjusted *P* values < 0.05. In the volcano plot, red dots indicate proteins positively associated with eGFR and *P* < 0.05. Blue dots indicate proteins negatively associated with eGFR and *P* < 0.05. Gray dots represent proteins that lack statistical significance in their association with eGFR.

Following the correlation analysis, regression analysis was performed to investigate the specific association of proteins (correlated with eGFR) with kidney function (Fig. 2). Among the correlated proteins, B2MG, FETUA, IGK, and VTDB showed a negative coefficient with eGFR of all participants, while A1AG2, CD44, CD59, CERU, KNG1, LV39, OSTP, PTGDS, REG1A, RNAS1, SH3L3, UROM, VMO1, and VTNC exhibited a positive coefficient with eGFR (Fig. 2A and Supplementary Table 3).

Furthermore, regression analysis was performed separately in CKD and control groups to identify the specific association of proteins (correlated with eGFR) with kidney function within each group. In CKD patients, correlated proteins such as B2MG, FETUA, IGK, AMBP, and VTDB showed a negative coefficient with eGFR, while LV39, CD59, A1BG, and CERU exhibited a positive coefficient (Fig. 2B). Among control participants, ATRN, SAP, and LG3BP protein showed a negative coefficient with eGFR, and only CRNN exhibited a positive coefficient with eGFR (Fig. 2C).

**Figure 3:**
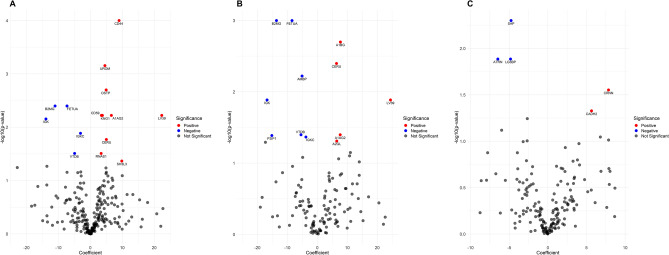
Volcano plot showing association between eGFR and emPAI of proteins adjusted for proteinuria. Regression analysis in all participants (**A**) and in patient (**B**) and control groups (**C**). On the X-axis, coefficient values show the relationship of individual proteins with eGFR, while the Y-axis conveys -log10-transformed *P* values for proteins with adjusted *P* values < 0.05. In the volcano plot, red dots indicate proteins positively associated with eGFR and *P* < 0.05. Blue dots indicate proteins negatively associated with eGFR and *P* < 0.05. Gray dots represent proteins that lack statistical significance in their association with eGFR.

Additionally, a regression analysis, adjusting for 24-h proteinuria, was performed to examine the association between eGFR and urinary proteins (Fig. 3). After adjustment for 24-h proteinuria, the correlated proteins B2MG, FETUA, IGK, and VTDB exhibited negative coefficients, while A1AG2, CD44, CD59, CERU, KNG1, LV39, OSTP, RNAS1, SH3L3, and UROM exhibited positive coefficients in the whole cohort (Fig. 3A and Supplementary Table 4).

In separate regression analysis, B2MG, FETUA, AMBP, and VTDB proteins consistently showed a negative coefficient with eGFR, while LV39, A1BG, and CERU exhibited positive coefficients in the patient group (Fig. 3B). In the control group, ATRN, SAP, and LG3BP protein consistently showed a negative coefficient with eGFR, and only CRNN consistently exhibited a positive coefficient with eGFR (Fig. 3C).

### Gene Ontology (GO) enrichment analysis

**Figure 4:**
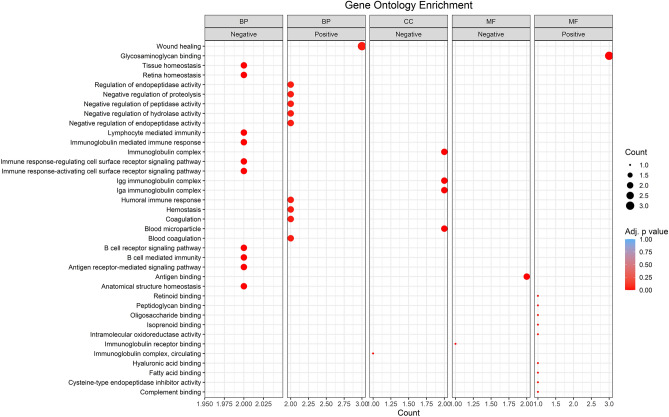
The top 10 terms for each GO category and associated proteins with kidney function. Each point represents a GO term listed on the left Y-axis. The size of the points corresponds to the count of proteins enriched in the respective term, and the color represents the adjusted *P* value significance. The plot is characterized by ontology categories, including biological process (BP), cellular component (CC), and molecular function (MF). The top 10 enriched terms for each category and the direction of negatively and positively associated proteins are highlighted.

The GO analysis based on biological processes showed that negatively associated proteins with eGFR demonstrated notable enrichment in retina homeostasis, tissue homeostasis, anatomical structure homeostasis, and immune response-related biological processes in the CKD group (Fig. 4). Conversely, positively associated proteins showed marked enrichment in the regulation and negative regulation of endopeptidase activity, negative regulation of proteolysis, negative regulation of hydrolase activity, negative regulation of peptidase activity, humoral immune response, hemostasis, and coagulation system processes in the CKD group.

The GO analysis based on cellular components exhibited prominently enriched blood microparticle, IgA immunoglobulin immunocomplex, and IgG immunoglobulin immunocomplex only in patients.

The GO analysis based on molecular functions showed that negatively associated proteins were enriched in antigen binding, while positively associated proteins exhibited enrichment in extracellular matrix structural constituent, glycosaminoglycan binding, in patients.

### Pathway enrichment analysis

Pathway enrichment analysis of the negatively and positively associated proteins with eGFR adjusted for proteinuria showed that negatively associated proteins were enriched in pathways related to the immune system, including neutrophil degranulation in patients (Table 4). Furthermore, immune system regulation-related pathways, such as regulation of the complement system, complement cascade, and homeostasis-related pathways, such as response to elevated platelet cytosolic Ca^2+^, platelet activation, signaling and aggregation, and fibrin clot pathways, were enriched by positively associated proteins in the CKD group. Also, the cellular adhesion pathway and integrin cell surface interactions were enriched by positively associated proteins in the CKD group (Table 4).

**Table 4:** The top 10 pathways of the positively and negatively associated proteins with eGFR adjusted for proteinuria.

Reactome pathways	Proteins	*P*-value	Adj. *p* value	Pathway type
Regulation of complement cascade	CD59, CLUS, IC1, VTNC	< 0.001	< 0.001	Positive
Complement cascade	CD59, CLUS, IC1, VTNC	< 0.001	< 0.001	Positive
Platelet degranulation	A1AG2, CLUS, IC1, KNG1	< 0.001	< 0.001	Positive
Response to elevated platelet cytosolic Ca^2+^	A1AG2, CLUS, IC1, KNG1	< 0.001	< 0.001	Positive
Platelet activation, signaling and aggregation	A1AG2, CLUS, IC1, KNG1	< 0.001	0.001	Positive
Integrin cell surface interactions	CD44, OSTP, VTNC	< 0.001	0.001	Positive
Post-translational protein phosphorylation	KNG1, CERU, OSTP	< 0.001	0.001	Positive
Insulin-like growth factor transport regulation by IGFBPs	KNG1, CERU, OSTP	< 0.001	0.002	Positive
Intrinsic pathway of fibrin clot formation	B2MG, KNG1	< 0.001	0.002	Positive
Formation of fibrin clot (clotting cascade)	B2MG, KNG1	< 0.001	0.004	Positive
Neutrophil degranulation	B2MG, FETUA	0.012	0.037	Negative
Nef mediated downregulation of MHC class I complex cell surface expression	B2MG	0.004	0.037	Negative
Endosomal/vacuolar pathway	B2MG	0.004	0.037	Negative
Vitamin D (calciferol) metabolism	VTDB	0.004	0.037	Negative
Nef-mediates down modulation of cell surface receptors by recruiting them to clathrin adapters	B2MG	0.008	0.037	Negative
Infection with *Mycobacterium tuberculosis*	B2MG	0.01	0.037	Negative
The role of Nef in HIV-1 replication and disease pathogenesis	B2MG	0.01	0.037	Negative
Antigen presentation: folding, assembly and peptide loading of class I MHC	B2MG	0.01	0.037	Negative
DAP12 signaling	B2MG	0.011	0.037	Negative

### Protein–protein interaction analysis

Among negatively associated proteins VTDB, showed interactions with B2MG and FETUA, while among positively associated proteins CERU, LV39, IC1, CLUS, VMO1, VTNC, CD59, REG1A, KNG1, CD44, SH3L3, OSTP, A1AG2, RNAS1, UROM, and PTGDS had complex interactions (Supplementary Fig. 1).

## Discussion

In this study, label-free quantitative proteomics was used to analyze the comprehensive 24-h urinary proteome of patients with early-stage CKD (stages 1–3) and healthy controls. Unlike prior research that often used spot urine samples and blood samples in CKD^12–14,25^, our approach offers a comprehensive perspective of the urinary proteome in early-stage CKD research. Also, we chose regression analysis adjusted for proteinuria over differential expression analysis to capture the nuanced relationships more accurately between urinary proteins and kidney function. This method allows for the consideration of covariates and predictive biomarkers, providing a comprehensive understanding of the underlying mechanisms involved. The analysis revealed positive and negative associations between kidney function and specific urinary proteins. Furthermore, the study identified distinct urinary protein profiles in CKD patients compared to healthy participants. Notably, proteins negatively associated with eGFR in CKD patients exhibited functional enrichment in processes related to tissue and structural homeostasis as well as immune system activity. Furthermore, positively associated proteins in CKD participants demonstrated significant enrichment in pathways related to extracellular matrix organization, cellular adhesion, coagulation, and the regulation of the immune system and enzyme activities.

Proteinuria is a complex pathophysiological process involving two common renal mechanisms: (i) abnormal protein excretion from the glomerular filter barrier, leading to glomerular proteinuria, and (ii) a disturbance in renal tubular handling of filtered proteins, causing tubular proteinuria. Heavy glomerular proteinuria places a significant strain on proximal tubular epithelial cells (PTECs). Alongside the toxic effects of proteinuria, this strain induces tubulointerstitial inflammation and fibrosis^11^. In routine clinical practice, only a few urinary proteins, such as kappa/lambda light chains and albumin, are employed as diagnostic markers. However, many other proteins remain undetectable and/or are not utilized in laboratory diagnostics.

We observed a substantially higher number of urinary proteins in healthy participants than in CKD patients (Table 1). Analyzing proteomes in complex biological samples is difficult, primarily because of the wide range of protein concentrations. In plasma, for instance, highly abundant proteins such as immunoglobulins and albumin, which can vary by over 10 orders of magnitude in concentration, can obscure the detection of low-abundant proteins, complicating the effectiveness of the MS method^26^. Similarly, the masking effect of highly abundant proteins can be observed when analyzing the urinary proteome of CKD patients, who have elevated levels of urinary albumin and other abundant proteins, making the identification of low-concentration urinary proteins challenging^27,28^.

Interestingly, our study's results align with prior research on the correlation patterns between protein molecular weight categories and renal markers. High molecular weight proteins (> 60 kDa), including KNG1, CD44, CERU, and UROM, exhibited a statistically significant positive correlation with eGFR in the whole cohort (Table 2). This consistency supports the notion that larger molecular weight proteins can serve as indicators of renal function^29–31^. Similarly, negative correlations of low molecular weight proteins, including FETUA, B2MG, IGK, KVD20, IGL1, VTDB, and IGHA1, with eGFR reflect trends observed in earlier studies^32–35^. On the other hand, low molecular weight proteins (< 60 kDa), such as VMO1, VTNC, CD59, REG1A, LV39, SH3L3, OSTP, A1AG2, RNAS1, and PTGDS, demonstrated positive correlations with eGFR in the whole cohort.

The regression analyses shed light on the complex relationship between urinary proteins and kidney function. Notably, proteins with negative coefficients, such as B2MG, FETUA, IGK, and VTDB, consistently exhibited associations with eGFR in the entire cohort, even after adjusting for proteinuria. Importantly, our further group-specific comprehensive analysis revealed that, among correlated proteins in the CKD group (Table 3), B2MG, FETUA, VTDB, and AMBP consistently demonstrated associations with eGFR, even after accounting for proteinuria (Figs. 2 and 3). These consistent negative associations raise the possibility regarding the role of these proteins as potential biomarkers for underlying kidney dysfunction.

In addition, the positively correlated proteins, including A1AG2, CD44, CD59, CERU, KNG1, LV39, OSTP, RNAS1, SH3L3, and UROM, exhibited robust associations with eGFR in the entire cohort, persisting even after adjustment for proteinuria. However, after group-specific analysis, only LV39, A1BG, and CERU, which were positively correlated with eGFR in the CKD group (Table 3), exhibited consistent associations with kidney function (Figs. 2 and 3). Persistent associations of these proteins suggest that elevated levels of these proteins in urine may reflect high or normal eGFR, indicating preserved kidney function. Exploring the clinical implications of these findings could pave the way for novel diagnostic approaches and therapeutic strategies.

The increase in low molecular weight proteins in urine, negatively associated with eGFR in patients, primarily reflects proximal tubular dysfunction and reduced tubular reabsorption^36^. Several studies have indicated the utility of urine B2MG levels in diagnosing tubular injury induced by sepsis, aminoglycosides, tenofovir, lithium, and heavy metals^33^. Also, elevated urinary B2MG levels have been linked to ongoing tubular dysfunction in adults with snake venom poisoning, persisting even after 6 months of eGFR recovery^37^, and were associated with lower eGFR after 1 year of acute kidney disease^38^. Besides, B2MG has been shown to induce oxidative damage to PTECs through the cadmium–B2MG complex and the FcRn–B2MG complex in proteinuric CKD^33,39^. Notably, an enrichment of an oxidative damage pathway was also reported in proteinuric patients, although this was not specifically linked to B2MG^40^. Another negatively associated protein, FETUA (fetuin-A), a hepatic secretory protein involved in various physiological processes, has emerged as another potential biomarker. Recent studies have reported an association between urinary FETUA and eGFR, suggesting its relevance in monitoring kidney function decline^34,41^. FETUA has also been found to protect kidneys from hypoxia-induced kidney damage, inflammation, and fibrosis^42^. Similarly, urinary AMBP (alpha-1-microglobulin/bikunin precursor) is a hepatic secretory protein readily filtered by the glomerulus and reabsorbed by PTECs. AMBP exhibits reductase, radical-scavenging, and heme-binding activities, protecting PTECs from oxidative damage by supporting mitochondrial function^43^. An increase in urinary AMBP levels has been noted as a marker of tubular dysfunction in patients with IgA nephropathy, diabetes and CKD^29,44^. VTDB (vitamin D-binding protein), the primary transporter protein of plasma vitamin D, has shown significant associations with eGFR and kidney function decline in studies on diabetic nephropathy and IgA nephropathy^29,32^. These consistent findings across different studies underscore the potential clinical significance of these proteins as indicators of kidney dysfunction and warrant further investigation into their diagnostic and prognostic utility. Our functional enrichment analysis results were also consistent with existing data that negatively associated proteins were predominantly associated with pathways involved in immune dysregulation and inflammatory response modulation corresponding to early-stage CKD pathology.

Among positively associated proteins with eGFR in patients, CERU (ceruloplasmin) is a high-molecular-weight protein and is responsible for 95% copper transport in the blood^45^. CERU was previously reported to correlate positively with eGFR in IgA nephropathy^29^. Normally, CERU (ceruloplasmin) is not freely filtered by the glomerulus. However, CERU can enter the tubule lumen during proteinuria and exert cytotoxic effects on PTECs under acidic conditions, thereby contributing to kidney pathology^46^. Other positively associated proteins, such as A1BG (alpha-1B-glycoprotein) and LV39 (immunoglobulin lambda variable 3–9) have received limited attention in the context of CKD, with few studies investigating their significance in this condition. A1BG, a plasma glycoprotein, previously was suggested as a marker to differentiate steroid-resistant nephrotic syndrome from non-resistant conditions in children^47^. Interestingly, in a recent study, A1BG was significantly upregulated in urine of snakebite patients with acute kidney injury (AKI)^48^. However, CKD and AKI have different causes, pathophysiological mechanisms, and progression, although there might be some overlap in biomarkers^49^. Overall, the functional enrichment of positively associated proteins with kidney function shows molecular mechanisms crucial for extracellular matrix organization, immune defense, and coagulation in CKD patients. The regulation of the extracellular matrix is essential to suppress its accumulation, prevent fibrosis formation, and maintain intercellular and ECM integrity. Importantly, proteinuria is a known contributor to renal inflammation and fibrosis by inhibiting the degradation of ECM components and inducing extracellular matrix synthesis, ultimately leading to ESKD^50^. Furthermore, the marked enrichment of platelet function-related pathways probably indicates increased platelet accumulation and activation due to glomerular injury to block blood loss after vascular damage. Activated platelets can interact with white blood cells and promote inflammatory kidney diseases^51,52^. Aggregates formed by platelet-white blood cell interaction were suggested to represent a marker of renal diseases and the prognosis of patients in a study by Finsterbusch et al.^52^. Therefore, the reduction of positively associated urinary proteins may contribute to the progression of CKD by affecting the immune system, blood clotting, and extracellular matrix organization pathways.

In the final step, interactions within proteins negatively and positively associated with eGFR were observed. VTDB exhibited the highest interactions among the negatively associated proteins. CLUS, IC1, KNG1, VTNC, CERU, OSTP, A1AG2, and UROM demonstrated the highest interactions among the positively associated proteins.

Our current findings build upon our pilot study, offering significant advancements in understanding urinary proteomics in CKD^35^. By including a larger sample size and meticulous data analysis, we have strengthened the reliability of our results. Unlike our pilot study, where we grouped participants based solely on proteinuria levels, this study used KDIGO guidelines, considering both eGFR and proteinuria. We also delved deeper into the association between the urinary proteome and eGFR, using correlation and regression analyses separately for the CKD and control groups. This provides a more comprehensive understanding compared to our previous study, which only used correlation analysis across the entire cohort due to its smaller sample size.

Despite the potential of urinary proteomics (using mass spectrometry) in identifying biomarkers for kidney disease, their limitations must be considered. The emPAI is a semiquantitative computational approach to estimate protein abundance and has inherent limitations and may not represent accurate protein abundance. Thus, validation of individual proteins using sensitive and specific immunoassays such as ELISA is needed to accurately quantify the protein abundance and confirm the reliability of the results. Furthermore, patients with early-stage CKD (1–3) were enrolled in this study. Therefore, they need follow-up to address alterations for the current findings and whether significantly associated proteins are early potential biomarkers of CKD progression. Next, the number of participants in the CKD groups is limited. Additionally, there was a gender imbalance between the patient and control groups. This imbalance may introduce potential confounding factors that could influence the interpretation of our results. Thus, a study with a larger sample size with more balanced gender representation in groups is needed to validate the current findings. During urine sample preparation for MS analysis, depletion of high-abundant proteins was not performed, limiting the identification of low-abundant urinary proteins in patients compared to controls. Finally, the glomerular disease diagnosis was not validated by kidney biopsy.

In this proteomics study, the type and number of urinary proteins differed substantially between patients in CKD stages 1–3 and healthy control participants. Specific urinary proteins demonstrated strong negative and positive associations with kidney function. Proteins with a negative association exhibited significant enrichment in pathways related to structural and tissue homeostasis and immune response. Proteins with a positive association exhibited significant enrichment in pathways related to extracellular matrix organization, cellular adhesion, coagulation, and the regulation of the immune system and enzyme activities in the early stages of CKD. Further validation studies are recommended to confirm the significance of each urinary protein associated with kidney function using immunoassay methods.

### Supplementary Information

Supplementary Information.

## Data Availability

The datasets generated and/or analyzed during the current study are available from the corresponding author on reasonable request.
