# Supplementary material for: Candidate protein biomarkers in chronic kidney disease: a proteomics study
Source: Sci Rep. 2024 Jun 18;14:14014. doi: 10.1038/s41598-024-64833-8 (PMC11189417; doi:10.1038/s41598-024-64833-8)
Supplement: Supplementary file 1 — Supplementary Information. [file 41598_2024_64833_MOESM1_ESM.docx]

**Candidate protein biomarkers in chronic kidney disease: a proteomics study**

**Supplementary materials**

**Table of contents**

**Supplementary Table 1.** Representative of participants by KDIGO CKD classification.

**Supplementary Table 2.** Average emPAI values of urinary proteins between groups.

**Supplementary Table 3.** Linear regression analysis between eGFR and emPAI of proteins.

**Supplementary Table 4.** Linear regression analysis between eGFR and emPAI of proteins adjusted for proteinuria.

**Supplementary Figure 1.** The protein network of associated proteins with kidney function.

**Supplementary Table 1.** Representative of participants by KDIGO CKD classification.


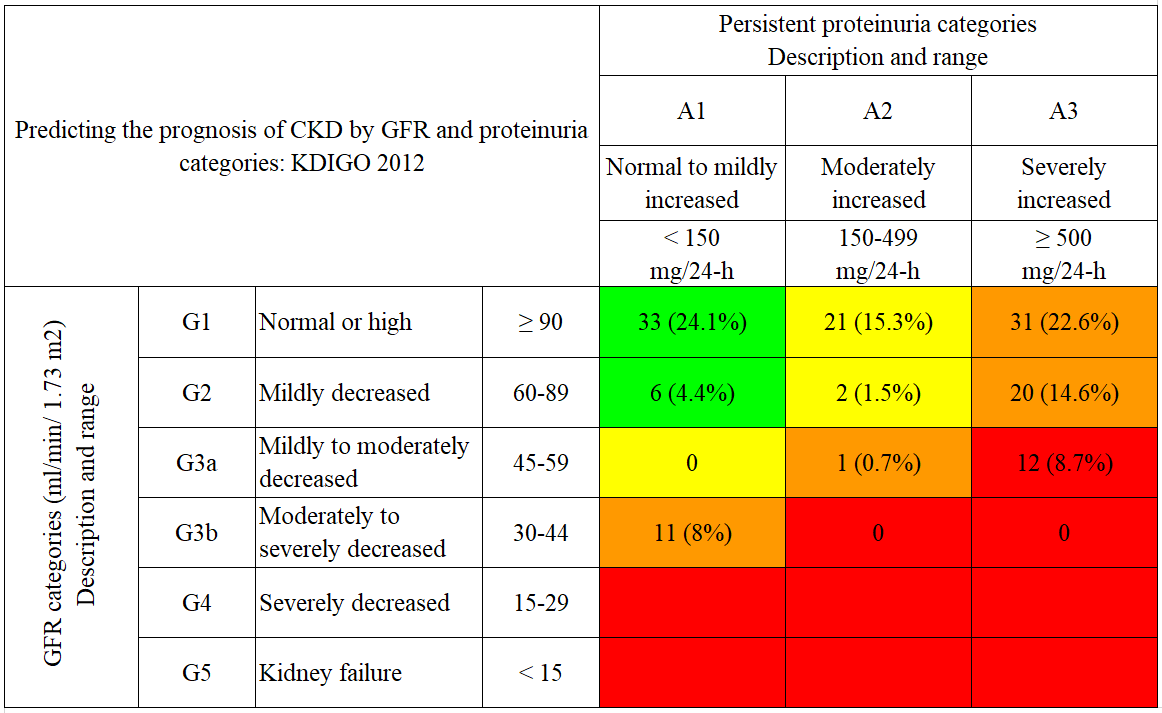


Green represents a low risk; yellow represents a moderately increased risk; orange represents a high risk; red represents a very high risk. CKD = chronic kidney disease; eGFR = estimated glomerular filtration rate.

**Supplementary Table 2.** Average emPAI values of urinary proteins between groups.

| **Urinary proteins** | **CKD group, median (IQR)** | **Control group, median (IQR)** |
| --- | --- | --- |
| FETUA | 0.25 (0.08–0.36) | 0.08 (0.08–0.16) |
| B2MG | 0.52 (0.23–0.53) | 0.24 (0.23–0.52) |
| AMBP | 1.47 (0.59–2.59) | 2.87 (1.33–5.34) |
| VTDB | 0.39 (0.19–0.64) | 0.19 (0.12–0.29) |
| IGK | 1.41 (1.12–2.09) | 0.89 (0.65–0.89) |
| IGKC | 5.92 (3.32–10.23) | 3.27 (1.36–4.47) |
| P3IP1 | 0.11 (0.11–0.23) | 0.37 (0.23–0.52) |
| LV39 | 0.26 (0.26–0.26) | 0.26 (0.26–0.26) |
| CD59 | 0.81 (0.49–1.47) | 2.41 (1.68–4.12) |
| A1BG | 0.84 (0.47–1.23) | 0.25 (0.18–0.28) |
| CERU | 0.16 (0.1–0.25) | 0.08 (0.05–0.1) |
| ATRN | 0.02 (0.02–0.02) | 0.04 (0.02–0.08) |
| SAP | 0.16 (0.1–0.16) | 0.11 (0.05–0.16) |
| LG3BP | 0.05 (0.05–0.05) | 0.15 (0.1–0.2) |
| PGRP1 | 0.14 (0.14–0.15) | 0.31 (0.031–0.49) |
| CRNN | 0.09 (0.06–0.12) | 0.06 (0.06–0.18) |
| A1AG2 | 0.64 (0.28–0.86) | 0.64 (0.45–0.87) |
| A2GL | 0.26 (0.17–0.37) | 0.17 (0.08–0.27) |

**Supplementary Table 3.** Linear regression analysis between eGFR and emPAI of proteins.

| **Proteome** | **Coefficient** | **95% CI** | ***P*-value** |
| --- | --- | --- | --- |
| LV39 | 22.0 | 6.4 – 37.6 | 0.007 |
| PI16 | 15.0 | 3.4 – 26.6 | 0.013 |
| IC1 | 13.8 | 8.4 – 19.2 | < 0.001 |
| PRIO | 12.7 | 2.0 – 23.3 | 0.022 |
| SH3L3 | 12.2 | 3.0 – 21.2 | 0.01 |
| CLUS | 10.7 | 2.4 – 19.0 | 0.014 |
| VTNC | 9.3 | 1.5 – 17.0 | 0.021 |
| VMO1 | 8.8 | 3.7 – 13.9 | 0.001 |
| CD44 | 8.2 | 4.5 –11.9 | < 0.001 |
| REG1A | 6.9 | 2.8 – 11.1 | 0.001 |
| HBB | 6.9 | 0.2 – 13.6 | 0.043 |
| LMAN2 | 6.2 | 0.4 – 11.9 | 0.034 |
| CD59 | 5.8 | 3.4 – 8.2 | < 0.001 |
| KNG1 | 5.64 | 3.4 – 7.9 | < 0.001 |
| A1AG2 | 5.6 | 1.3 – 9.9 | 0.011 |
| OSTP | 5.3 | 2.5 – 8.0 | < 0.001 |
| CERU | 5.1 | 1.2 – 8.9 | 0.011 |
| RNAS1 | 4.6 | 1.8 – 7.4 | 0.001 |
| PIGR | 4.4 | 0.2 – 8.6 | 0.04 |
| FBN1 | 4.3 | 0.8 – 7.9 | 0.017 |
| PTGDS | 4.1 | 0.9 – 7.3 | 0.011 |
| UROM | 3.7 | 1.7 – 5.6 | < 0.001 |
| A1AG1 | 3.5 | 0.1 – 7.0 | 0.045 |
| IGK | -14.3 | -24.1 – 4.5 | 0.005 |
| B2MG | -11.2 | -18.5 – 3.8 | 0.004 |
| FETUA | -8.2 | -12.8 – 3.5 | 0.001 |
| VTDB | -5.6 | -9.8 – -1.3 | 0.011 |
| IGKC | -3.3 | -5.7 – -0.8 | 0.01 |

CI = confidence interval.

**Supplementary Table 4.** Linear regression analysis between eGFR and emPAI of proteins adjusted for proteinuria.

| **Proteome** | **Coefficient** | **95% CI** | ***P*-value** |  |
| --- | --- | --- | --- | --- |
| LV39 | 22.4 | 6.9 – 37.9 | 0.006 | |
| SH3L3 | 9.8 | 0.3 – 19.3 | 0.043 | |
| CD44 | 9.0 | 5.0 – 12.0 | < 0.001 | |
| A1AG2 | 6.6 | 1.9 – 11.2 | 0.006 | |
| CERU | 5.0 | 0.9 – 9.1 | 0.017 | |
| OSTP | 4.9 | 1.8 – 8.0 | 0.002 | |
| UROM | 4.5 | 2.1 – 7.0 | < 0.001 | |
| KNG1 | 3.8 | 1.1 – 6.4 | 0.006 | |
| CD59 | 3.4 | 1.0 – 5.9 | 0.006 | |
| RNAS1 | 3.4 | 0.3 – 6.4 | 0.031 | |
| IGK | -13.9 | -23.9 – -4.0 | 0.007 | |
| B2MG | -11.1 | -18.4 – -3.7 | 0.004 | |
| FETUA | -7.3 | -12.1 – -2.5 | 0.004 | |
| VTDB | -4.9 | -9.4 – -0.5 | 0.031 | |
| IGKC | -3.1 | -5.5 – -0.6 | 0.013 | |

CI = confidence interval.

| **A** | | **B** | |
| --- | --- | --- | --- |
| 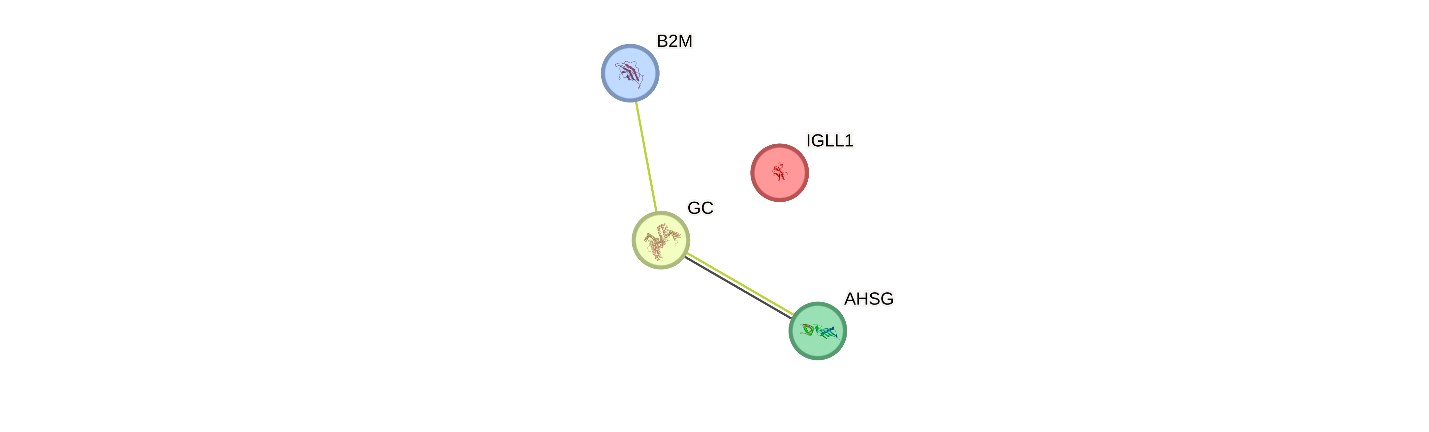 | 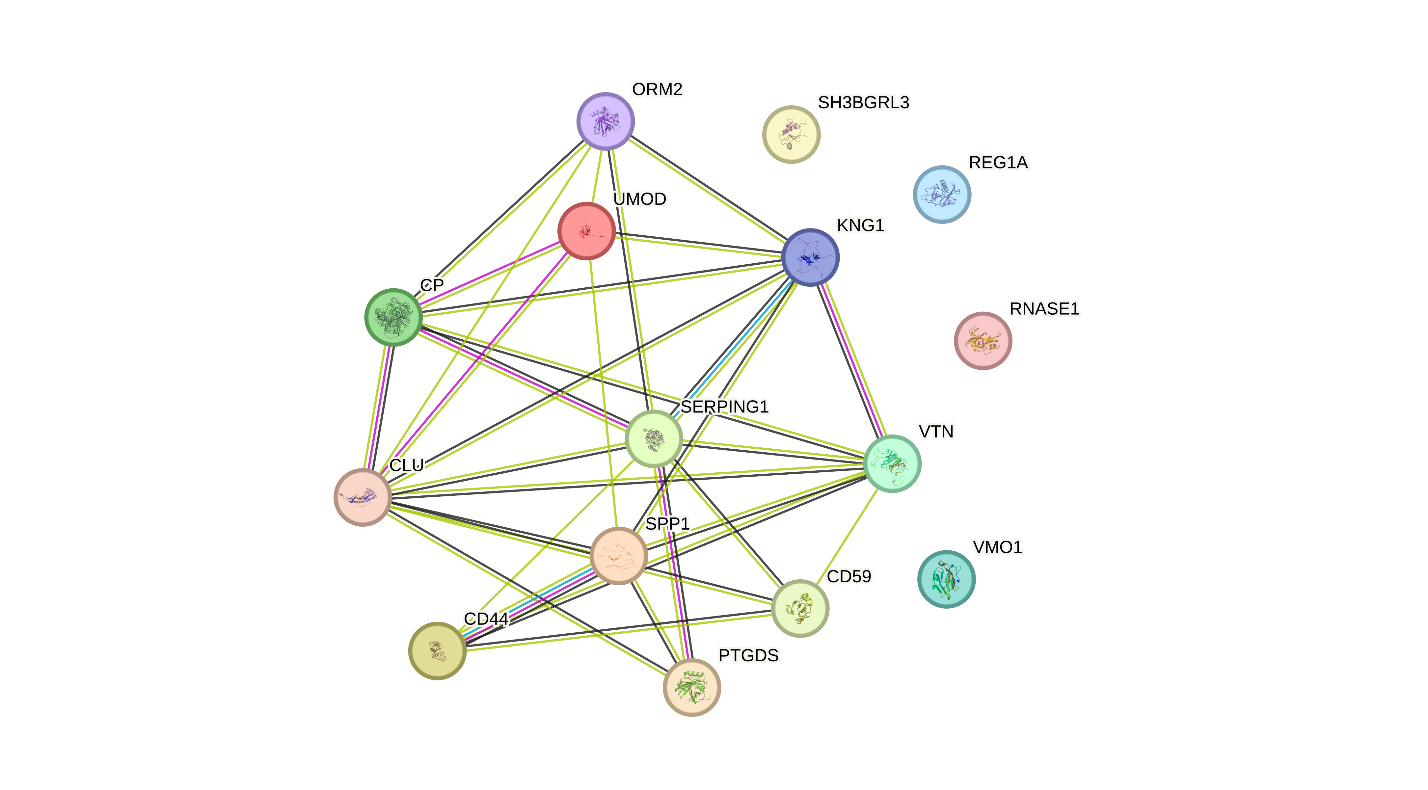 | |  |

**Supplementary Figure 1.** The protein network of associated proteins with kidney function. The protein network of negatively associated proteins (**A**) and positively associated proteins (**B**) with eGFR adjusted for proteinuria is depicted. The nodes in the network represent proteins, and the edges represent interactions between proteins. Gene names of corresponding proteins were used as input.
